# Barriers, frameworks, and mitigating strategies influencing the dissemination and implementation of health promotion interventions in indigenous communities: a scoping review

**DOI:** 10.1186/s13012-022-01190-y

**Published:** 2022-02-21

**Authors:** Lea Sacca, Ross Shegog, Belinda Hernandez, Melissa Peskin, Stephanie Craig Rushing, Cornelia Jessen, Travis Lane, Christine Markham

**Affiliations:** 1 Center for Health Promotion and Disease Prevention, University of Texas Health Science Center at Houston School of Public Health, 7000 Fannin, Houston, TX 77030 USA; 2 Center for Health Promotion and Disease Prevention, University of Texas Health Science Center School of Public Health in San Antonio, 7411 John Smith Drive, Suite 1100, San Antonio, TX 78229 USA; 3 Northwest Portland Area Indian Health Board, 2121 SW Broadway Suite 300, Portland, OR 97201 USA; 4 Alaska Native Tribal Health Consortium, 4000 Ambassador Drive, Anchorage, AK 99508 USA; 5 Inter Tribal Council of Arizona, Inc., 2214 North Central Avenue, Phoenix, AZ 85004 USA

**Keywords:** Dissemination frameworks, Implementation barriers, Indigenous communities, SISTER strategies, Cultural context

## Abstract

**Background:**

Many Indigenous communities across the USA and Canada experience a disproportionate burden of health disparities. Effective programs and interventions are essential to build protective skills for different age groups to improve health outcomes. Understanding the relevant barriers and facilitators to the successful dissemination, implementation, and retention of evidence-based interventions and/or evidence-informed programs in Indigenous communities can help guide their dissemination.

**Purpose:**

To identify common barriers to dissemination and implementation (D&I) and effective mitigating frameworks and strategies used to successfully disseminate and implement evidence-based interventions and/or evidence-informed programs in American Indian/Alaska Native (AI/AN), Native Hawaiian/Pacific Islander (NH/PI), and Canadian Indigenous communities.

**Methods:**

A scoping review, informed by the York methodology, comprised five steps: (1) identification of the research questions; (2) searching for relevant studies; (3) selection of studies relevant to the research questions; (4) data charting; and (5) collation, summarization, and reporting of results. The established D&I SISTER strategy taxonomy provided criteria for categorizing reported strategies.

**Results:**

Candidate studies that met inclusion/exclusion criteria were extracted from PubMed (*n* = 19), Embase (*n* = 18), and Scopus (*n* = 1). Seventeen studies were excluded following full review resulting in 21 included studies. The most frequently cited category of barriers was “Social Determinants of Health in Communities.” Forty-three percent of barriers were categorized in this community/society-policy level of the SEM and most studies (*n* = 12, 57%) cited this category. Sixteen studies (76%) used a D&I framework or model (mainly CBPR) to disseminate and implement health promotion evidence-based programs in Indigenous communities. Most highly ranked strategies (80%) corresponded with those previously identified as “important” and “feasible” for D&I The most commonly reported SISTER strategy was “Build partnerships (i.e., coalitions) to support implementation” (86%).

**Conclusion:**

D&I frameworks and strategies are increasingly cited as informing the adoption, implementation, and sustainability of evidence-based programs within Indigenous communities. This study contributes towards identifying barriers and effective D&I frameworks and strategies critical to improving reach and sustainability of evidence-based programs in Indigenous communities.

**Registration number:**

N/A (scoping review)

**Supplementary Information:**

The online version contains supplementary material available at 10.1186/s13012-022-01190-y.

Contributions to the literature

- Informs and guides future D&I initiatives aimed at reducing health disparities in Indigenous communities
- Identifies common D&I barriers that appear salient for Indigenous communities
- Identifies effective mitigating D&I models and strategies to successfully disseminate and implement evidence-based programs in American Indian/Alaska Native (AI/AN), Native Hawaiian/Pacific Islander (NH/PI), and Canadian Indigenous communities
- Informs the development of culturally tailored D&I strategies to improve efforts to scale-up effective interventions among Indigenous communities

## Background

Many Indigenous communities across the USA and Canada experience a disproportionate burden of health disparities [1–3]. These disparities exist across populations, age ranges, public health domains, disease prevention, and management contexts. For example, American Indian/Alaska Native (AI/AN) and Native Hawaiian/Pacific Islander (NH/PI) youth, in particular, have experienced higher prevalence of sexual and reproductive health and chronic disease disparities [1–3]. In 2017, AI/AN females (15-19 years) had the highest teen birth rate (32.9 per 1000) compared to other racial/ethnic groups (18.8 per 1000) nationally [3]. Further, compared to white peers, AI/AN and NH/PI youth exhibit higher prevalence of obesity (76.7% vs. 63.2%), diabetes (21.4% vs. 8%), and mental health conditions (including a 3-fold greater suicide rate) [4]. Similarly, prevalence of diabetes in Canadian First Nations and Inuit communities is 2.5 to 5 times greater than the general population [5], and First Nations communities experience higher rates of cancer due to limited access to preventive services [2, 6, 7]. In response, Indigenous communities have partnered with researchers to design and evaluate culturally relevant health programs. This work has increased the availability of a number of evidence-based interventions (EBIs) suitable for implementation in Indigenous communities [8–32].

Evidence-based interventions (EBI) refer to treatments that have been evaluated for a degree of effectiveness in changing target behavior through outcome evaluations [33, 34]. They are validated for a specific purpose when applied to a specific population and thus are only useful for a range of health and social problems that underly its design [34]. Changing parts of the EBI will invalidate it by impacting its integrity and effectiveness [34]. Validation of EBIs occurs through large group research or a series of small group studies [33, 34]. However, there might be cases where the intervention was not effective when applied to a specific case [34]. The use of mainstream “evidence-based practices” (EBP), in place of culturally relevant programs, has been a subject of concern in Indigenous communities—where the use of EBP are mandated by Federal or State funding—conflicting with tribal values or ways of knowing [35–39]. Evidence-based public health practices involve the development, implementation, and evaluation of effective programs and policies in public health through the utilization of principles of scientific reasoning to combine individual clinical expertise with the most prominent scientific evidence [40, 41]. It draws on principles of good practice and integrates sound professional judgments with a systematic body of research [42]. Emergent practices, including practice-based evidence and cultural adaptation can improve the compatibility of EBPs in AI/AN communities [33]. Indigenous tribes and researchers have advocated for the inclusion of traditional practices in evidence-based programs [35, 36, 43], and Tribal Best Practices (TBP) have bridged that divide, incorporating both cultural-based evidence and testable outcomes [33].

The design of culturally relevant EBPs in Indigenous communities ranges from surface to deeper level adaptations [37]. Few mainstream EBPs have been rigorously evaluated with AI/AN populations, which in turn generates limited outcomes or impacts for this group [44–46]. Some EBPs may be better aligned with tribal usability and acceptability than others [46]. There exists a need to further explore EBIs, EBPs, and evidence-informed programs (EIPs) in the context of Indigenous populations [33, 43, 46, 47]. Evidence-informed programs (EIPs), a sub-category of EBIs, are of particular interest—as they aim to integrate research evidence, alongside practitioner expertise, as well as community members’ experience with the practice—such as elders, adults, children, community-health workers, and tribal leaders [48–50].

The emergence of EBPs, cultural adaptations, and their associated evidence base increases the importance of understanding the most salient barriers and facilitators to the successful adoption, implementation, dissemination, and sustainability of EBIs in Indigenous communities. Several contextual factors can assist or hinder this process and may be further confounded by the geographic, cultural, and political diversity of Indigenous communities [9]. These factors can occur at each level of the socio-ecological model (SEM) [8–32]. Individual (intrapersonal) factors include characteristics, attitudes, and skills of program staff to implement and evaluate programs. Interpersonal factors include influencing roles of family members, peers, and mentors and their training skills. Organizational factors include administrative support, cultural components, and management of resources within Indigenous organizations (e.g., staff turnover and training, participant recruitment and retention, technology availability and use, program funding). Community factors are embedded within the physical and social environment (e.g., integration with cultural values, transportation). Public policy factors include social and cultural norms supporting certain behavioral outcomes, along with health, educational, economic, and social policies that exacerbate social inequalities between subgroups in Indigenous communities [11]. The requirements and demands of implementing EBIs are often mismatched with the capacities of the Indigenous communities that need them, undermining broad EBI scale-up and dissemination [51]. Increased reach and implementation of EBIs can be facilitated by the use of guiding dissemination and implementation (D&I) frameworks, theories, and models, referred hereto as models [52, 53] and by the application of empirically validated strategies [54, 55]; yet few studies have examined their application in guiding the implementation of EBIs within Indigenous communities [8, 10]

### Dissemination and implementation models

The formalization of research in D&I is growing and numerous models exist to guide this process [52, 53]. Research-to-practice models are most frequently applied and are intended for use by diverse stakeholders (e.g., researchers, community-based practitioners, and funders) to systematically guide and critically assess prevention efforts [56, 57]. They also help to inform on specific D&I steps, such as community needs assessment, to identify important barriers and facilitators, and inclusion of community members’ expert knowledge in implementation planning, and assessment of community capacity [56]. The “Dissemination and Implementation Models in Health Research and Practice Webtool,” a collaboratively developed decision support tool, provides an updated database of D&I frameworks to assist researchers and practitioners to generate research questions, select, adapt, and combine D&I models for particular study contexts, and implement and evaluate D&I models [53]. Despite the utility of D&I models and availability of decision tools, their application to guide program implementation has been the exception rather than the rule [8, 9, 58, 59].

### Implementation strategies

These are practical tasks (often associated with D&I models) recommended to aid the successful D&I of research findings into clinical and community practice [60]. Taxonomies of strategies to successfully facilitate the adoption, use, and maintenance of EBIs include the ERIC (Expert Recommendations for Implementing Change) and SISTER (School Implementation Strategies, Translating ERIC Resources) taxonomies [54, 55]. The ERIC taxonomy comprises 73 strategies devoted to implementation of EBIs in healthcare settings [54, 60]. The SISTER strategies are an adaptation from those in ERIC but focused on, and more compatible with, school and community-based contexts [61]. The SISTER taxonomy comprises nine domains: (1) use evaluative and iterative strategies; (2) provide interactive assistance; (3) adapt and tailor to context; (4) develop stakeholder interrelationships; (5) train and educate stakeholders; (6) support educators; (7) engage consumers; (8) use financial strategies; and (9) change infrastructure [59, 60]. Within the nine domains are 75 strategies focused on training, local technical assistance, adoption, high fidelity implementation of EBIs, and program replication in school-based settings [62, 63]. Additional previously identified strategies, seminal to use in Indigenous communities, include integration of EBIs within the cultural context [64, 65], involvement of Indigenous leaders, and ensuring sufficient resources (i.e., economic, health, and political) [9, 64, 65].

The purpose of this scoping review was to identify common barriers and effective mitigating D&I models and strategies to successfully disseminate and implement evidence-based programs in American Indian/Alaska Native (AI/AN), Native Hawaiian/Pacific Islander (NH/PI), and Canadian Indigenous communities. This review builds on a published multi-case study by Jernigan et al. (2020) to develop culturally tailored D&I strategies to enhance the ability of researchers to scale up effective interventions among Indigenous communities [8]. This scoping review may further contribute to informing and guiding future D&I initiatives aimed at reducing health disparities in this population.

## Methods

The review team comprised researchers with expertise in D&I and in the development and implementation of EBIs for Indigenous communities in the US and Canada. The PRISMA-ScR (Preferred Reporting Items for Systematic reviews and Meta-Analyses extension for Scoping Reviews) was used as a reference checklist in the development of the study sections [66]. Arksey and O’Malley’s (2005) York methodology guided the review [67]. This framework methodology comprises five steps to (1) identify research questions; (2) search for relevant studies; (3) select studies relevant to the research questions; (4) chart the data; and (5) collate, summarize, and report results. The method ensures transparency, enables replication of the search strategy, and increases the reliability of study findings [67].

### Step 1. Identify research questions

Three guiding research questions for the scoping review were: (1) What are the main barriers encountered in the D&I of programs and EBIs in Indigenous communities?; (2) Which research-to-practice models have been used to promote the D&I of health promotion EBIs in Indigenous communities?; (3) What implementation strategies have been used in Indigenous communities for program and EBI adoption, implementation and/or maintenance?

### Step 2. Search for relevant studies

Keywords and mesh terms were developed in corroboration with a research librarian experienced with scoping review protocols. Search terms focused on AI/AN and NH/PI communities, Native communities, Indigenous tribes, tribal groups, dissemination models, dissemination frameworks, implementation frameworks, EBIs, and US and Canadian territories (Table 1). Educational subject headings and Boolean operators were adopted as search tools to narrow, widen, and combine literature searches. The Rayyan platform was used to condense all studies generated from our search [68]. Three electronic databases (PubMed, EMBASE, and Medline (Ovid)), selected for their breadth and focus on psychosocial and behavioral science, were searched to identify peer-reviewed literature from primary data sources, secondary data sources, and case reports. The review of the literature databases was completed over a period of 2 months, ending in June 2020. Articles were screened for eligibility by reviewer pairs (CM and BH; RS and MP) over a period of 3 months, ending in September 2020.

**Table 1:** Key search terms

Keywords	Mesh terms
Dissemination^a^	Information dissemination; dissemination; diffusion of innovation; health information exchange; health information management; Public health surveillance; informatics; information management
Implementation^b^	Implementation; health plan implementation; implementation science; regional health planning; social planning
Assessment	Process assessment; process measures
AI/AN; NH/PI communities	Tribes; natives; native-born; American Indian; Alaska Native; Native Hawaiian; Pacific Islander; Indigenous populations; Indigenous communities; Canadian aboriginals
Interventions	Interventions; preventive health services; programs; health promotion programs

^a^Dissemination is the distribution of intervention information and material to a specific public community or clinical practice audience (defined by the National Institute of Health) [58]

^b^Implementation is the utilization of strategies to adopt and integrate evidence-based health interventions within specific settings (defined by the National Institute of Health) [58]

#### Inclusion criteria

Included were peer-reviewed studies, published in English between 2000 and 2020 that (1) described the use of D&I models and frameworks to increase the dissemination, implementation, or maintenance of evidence-based or evidence-informed programs among Indigenous communities, and (2) were conducted among AI/AN, NH/PI, and Indigenous populations of any age range located in the USA or Canada. ‘Dissemination’ and ‘Implementation’ were defined in accordance with the 2016 National Institute of Health definitions [69]. Indigenous populations of interest included individuals identifying as AI/AN, NH/PI, or Indigenous in the USA and Canada. EBIs were defined as any evidence-based or evidence-informed intervention or program disseminated or implemented in AI/AN, NH/PI, and/or Canadian Indigenous communities to improve health and behavioral outcomes. The rigor of evidence supporting the dissemination, implementation, or maintenance of these programs was not a criterion by which articles were included or excluded. Articles that describe the D&I of either evidence-based or evidence-informed programs were included.

#### Exclusion criteria

**Fig. 1:**
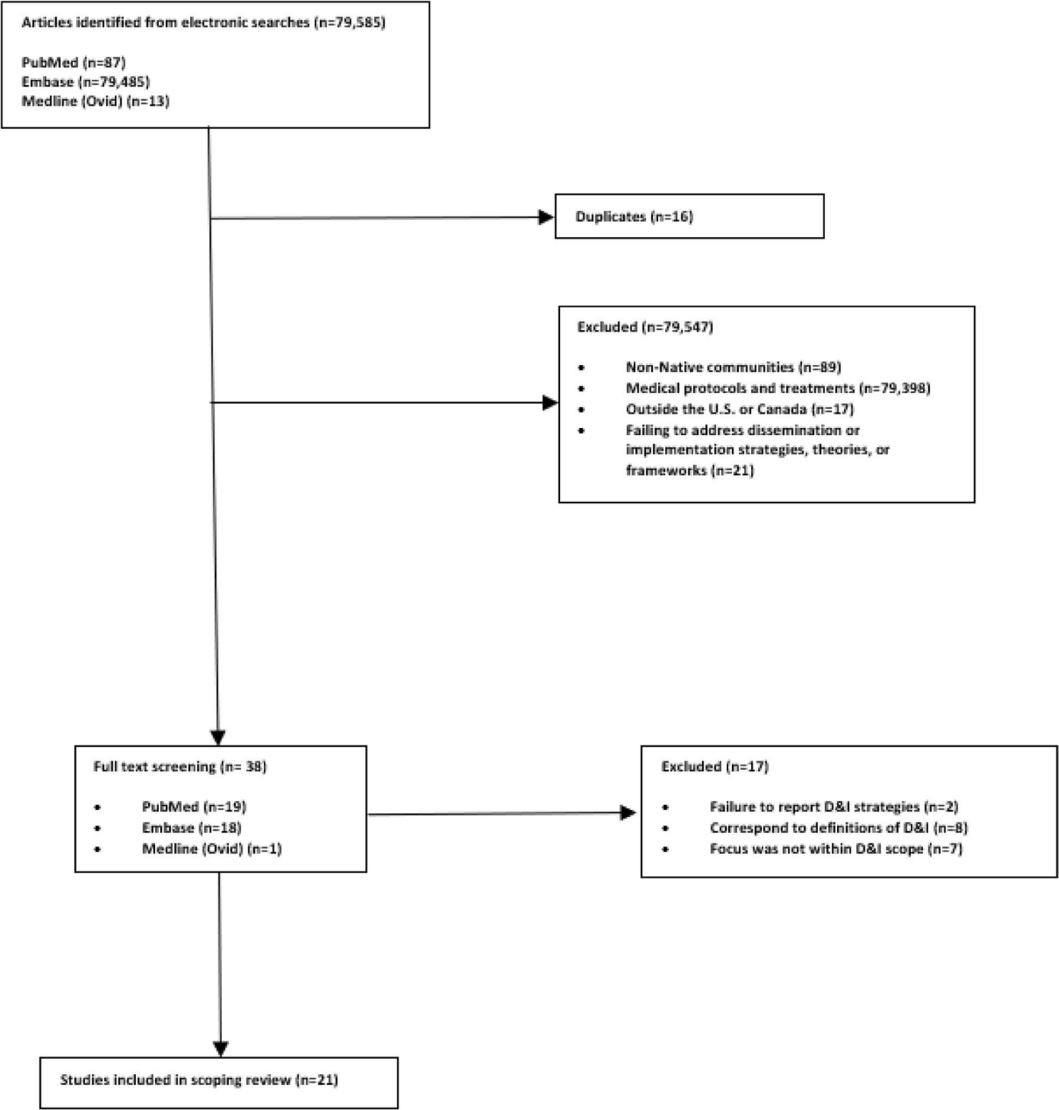
Flow chart of the study selection process

Excluded were studies that addressed populations distinct from Indigenous communities or targeted samples that did not exclusively identify as Indigenous communities located in the USA or Canada, studies focusing solely on improved behavioral or health outcomes with no reference to the D&I field, and studies that only reported general recruitment strategies, follow-up studies after the implementation of a program, or that focused solely on ethical issues related to the implementation of these programs. Initial screening and Rayyan page construction were performed by the lead author (LS). Reviewer pairs (CM and BH; RS and MP) conducted secondary screening of the titles and abstracts. Disagreements were resolved by reaching consensus through discussions that involved the initial reviewer (LS) (Fig. 1).

### Step 3. Selection of studies relevant to the research questions

The lead author (LS) extracted and summarized the data from relevant studies. Reviewer pairs (CM and BH; RS and MP) reviewed the data extraction and summary tables for accuracy. Conflicting opinions were resolved by consensus discussion. Summary tables included an evidence table describing each study’s parameters including guiding D&I models, identified barriers, and mitigating strategies. D&I models were identified using the ‘Dissemination and Implementation Models in Health Research and Practice Webtool’ previously described [53]. Barriers, contextual factors that hinder implementation at each level of the socio-ecological model (SEM) [11], were classified by the 5 levels of the (SEM) and by barrier categories based on major themes within the broader SEM framework. The SEM framework acts as a comprehensive external reference to the D&I models and strategies; therefore, it aids in the assessment of such models and strategies when applied to multiple and interacting determinants of health behaviors [11].

D&I strategies were categorized and coded according to the SISTER framework (previously described). The SISTER taxonomy was used as the referent due to its utility for school and community-based contexts [61]. Initial categorization and coding by the lead author (LS) was compared to independent categorization with reviewer pairs for inter-rater reliability in a subsample of 38% (*n* = 8) studies. Inter-rater reliability was conducted in two rounds with discrepancies resolved by consensus discussion. Resulting inter-rater reliability was 90% for strategy-level matching and 70% for domain-level matching (Supplemental Tables 1 & 2).

### Steps 4 and 5. Data charting and collation, summarization, and reporting of results

Study characteristics were tabulated for primary author, country, study type, sample size, target population, study topic area, and D&I model (Table 2). Identified barriers were tabulated by SEM level and classified to one of nine barrier categories (Personnel Challenges & High Turnover; Distrust; Funding; Lack of Integration with Cultural Values; Social Determinants of Health in Communities (physical, mental, health, social, and financial challenges); Insufficient Evaluation Skills; Technology Barriers; Limited Retention and High Attrition; Climate Conditions) (Table 3). The specific strategies were rank ordered within the SISTER domains, as well as based on importance and feasibility (Table 4).

**Table 2:** Study characteristics

#	Author/country	Study design	Sample (size)	Priority population	Stakeholders	Intervention/program topic area	D&I theory/framework
1	Barlow (2018) [16] (USA)	Case study	Choctaw (*n* = 220,000), Apache (*n* = 17,000), Kodiak (*n* = 226), & Native American Health Center (*n* = 7,200)	AI/AN mothers and infant caregivers	Indigenous home visitors; Staff from Urban Indian Center	Evaluation of the Tribal Maternal and Early Childhood Home Visiting (MIECHV) legislation supporting the delivery of home-visiting interventions in low-income AI/AN communities	None
2	Black (2018) [17] (USA)	Randomized controlled trial	AI/AN youth from program delivery sites in tribal communities (*n* = 16)	AI/AN youth	Tribal partners (funding agencies, academic institutions); Chief program officers; Program staff; Community advisory group	Implementation of a sexual health intervention for AI/AN youth.	CBPR
3	Jernigan (2020) [20] (USA)	Case study series	Community-based organization on major Hawaiian Islands (*n* = 30) (KaHOLO Project); indigenous adolescents (*n* = 200) across 10 urban communities across California (MICUNAY); 1,640 shoppers from Chickasaw Nation and Choctaw Nation of Oklahoma (THRIVE Study)	Native Hawaiians at risk of CVD and HT (KaHOLO Project); Urban Native American Youth (Motivational Interviewing and Culture for Urban Native American Youth-MICUNAY); shoppers from Chickasaw Nation and Choctaw Nation of OK (THRIVE Study)	Hula community; Native Hawaiian Health Task Force; Community members; Health care providers; Tribal government; Commerce; Health sectors	Assessment of three D&I case studies of NIH-funded intervention research to improve Native American Health (IRINAH)	CBPR (KaHOLO Project & MICUNAY); Reach, Efficacy, Adoption, Implementation, & Maintenance (RE-AIM) Framework (THRIVE study)
4	Counil (2012) [13] (Canada)	Qualitative	5 participants (Inuk leader; Inuk student; southern student; southern nutritionist; and southern researcher)	Inuit communities in Greenland & Northern Canada	Inuk leader; Inuk student; southern student; southern nutritionist; and southern researcher	Implementation of a reduction of the trans-fat content of food sold in Nunavik	None
5	Craig Rushing [12] (2018) (USA)	Pilot	50 states and 73 countries	AI/AN youth	Representatives from community-based organizations; Tribal health educators; advocates; teachers; school counselors; university partners	Assessing the reach and usability of the Healthy Native Youth website including culturally acceptable sexual health curricula	None
6	Douglas (2013) [18] (Canada)	Pilot	First Nation children with asthma and their caregivers (*n* = 13)	First Nation children with asthma in Canada	National advisory group; instructors; health professionals; academics with expertise in asthma education	Adaptation of the “Roaring Adventures of Puff Program” for First Nation Children with asthma	Knowledge-to-Action Framework
7	Gates (2013) [19] (Canada)	Case study	First Nations youth attending one school in Kashechewan, Ontario (sample size not specified)	First Nations youth	School administrators; university researchers; community key stakeholders	Lessons learned following the implementation of a school-based snack program for Native Youth	CBPR
8	Jernigan (2016) [20] (USA)	Cross-sectional	Key stakeholders in Oklahoma (*n* = 100) and California (*n* = 75)	AI stakeholders in two reservations (California and Oklahoma)	Community advisory board; university research center	Assessing obesity through policy and environmental approaches in two AI communities	CBPR
9	Jiang (2013) [21] (USA)	Quasi-experimental	Participants from AI/AN communities (*n* = 2,553)	80 AI/AN tribes served by 36 healthcare programs	IHS-contracted health programs; IHS hospitals/clinics; lifestyle coaches	Evaluation of the special diabetes program for Indians Diabetes Prevention	CBPR
10	Kaufman (2018) [22] (USA)	Cross-sectional	Stakeholders involved with sexual health and well-being of AI/AN youth (*n* = 142)	AI/AN youth	Expert task force (local technicians, CDC, IHS personnel, experts in HIV/STD)	Identification and assessment of the parameters facilitating the uptake of a sexual risk reduction EBI (RESPECT)	Diffusion of Innovation
11	Markham (2016) [10] (USA)	Randomized controlled trial	AI/AN youth (12-14 yrs.) from 13 urban (*n* = 13) & rural/tribal (*n* = 12) settings in AK, AZ, OR, ID, WA.	AI/AN youth	Regional staff; site coordinators (teachers, counselors, nurses, wellness coordinators, and college students)	Assessing the impact of the internet in the delivery of evidence-based health programs	None
12	Martindale-Adams (2017) [23] (USA)	Randomized controlled trial	Caregiving dyads from a federal or Tribal health care program serving one of the 546 federally recognized Tribes, an Urban Indian Health program, or awardees of the ACL/AOA Native American Caregiver Support Program (NACSP)	AI/AN with Alzheimer’s disease or early dementia	Staff from tribal healthcare programs; public health nurses; community health representatives; university research center	Implementation of REACH (Resources for Enhancing Alzheimer’s Caregivers Health) for an EBI Alzheimer’s EBI	Implementation Process Model
13	Mokuau (2008) [24] (USA)	Qualitative	Native Hawaiian elders seeking health services at the National Resource Center established at the University of Hawaii	Native Hawaiian elders	University of Hawaii research center; congressional leaders; national leaders in Native elder health; leaders at the University of Hawaii; gerontologists; Native Hawaiian leaders in the community	Development of a National Resource Center for Hawaiian elders to decrease disparities in accessing health services	CBPR
14	Moleta (2017) [25] (USA)	Quasi-experimental	Community Health Workers (CHWs) (*n* = 46)	Community Health Workers in Native communities	Ulu network members; Center for Native and Pacific Health Disparities Research	Development, Implementation, and Evaluation of “Heart 101”, a cardiovascular disease training program in Hawaii	CBPR/Adult Learning Theory
15	Nadin (2018) [26] (Canada)	Quasi-experimental	7 client and family members; 22 healthcare providers	First Nation elderly people	Community care program staff; federal and provincial government; funding agencies; external resources; healthcare providers; elders; members of the Band council and administration	Process evaluation of a pilot implementation of a community-based palliative care program (Wiisokotaatiwin)	CBPR
16	Orians (2004) [15] (USA)	Multisite case study design	141 interviews with key informants and 16 focus groups (132 AI/AN eligible women)	AI/AN eligible women	Program site staff; tribal members; health educators; outreach workers	Assessment of the tribal programs’ implementation of the public education and outreach component of CDC’s National Breast and Cervical Cancer Early Detection Program	CBPR
17	Pei (2019) [28] (USA)	Qualitative	35 participants in the Parent-Child Assistance Program for fetal alcohol spectrum disorder	First nation communities enrolled in fetal alcohol spectrum disorder services	First Nation community; leaders; program staff; university research members	Assessment of mentors' perceptions of the impacts and suitability of a relational, trauma-informed, and community-based approach to service delivery in First Nation communities	CBPR
18	Rasmus (2019) [29] (USA)	Case Study	Alaska Native communities suffering from the burden of suicide and alcohol misuse (sample size not specified)	AN communities	Indigenous researchers; Zuni tribal members and teachers; local community advisory; advisory committee; tribal/university collaboration; elders	Development of an Indigenous knowledge theory-driven intervention to guide researchers in indigenous communities who seek to create Indigenously informed and locally sustainable strategies for the promotion of health and well-being	Theory of Change framework/Indigenous Knowledge and Cultural Logic Model of Contexts
19	Short (2014) [30] (Canada & USA)	Systematic review	10 Indigenous communities suffering from motor vehicle crashes (MVC)	Indigenous communities	Child restraint technicians; police officers; prenatal and child safety seat clinic staff; Head Start staff	Successful dissemination and implementation strategies used in the development and implementation of MVC interventions	None
20	Walters (2020) [31] (USA)	Case study series	Yappalli Choctaw Study: Choctaw women (sample size not specified); the Qungasvik (Toolbox) Prevention Approach: AN youth 12–18 years old (sample size not specified); KaHOLO Project: Native Hawaiian adults at risk of cardiovascular disease and hypertension (sample size not specified)	Native communities	Choctaw health leaders; non-Native support staff; Native allies; Choctaw community members; community and cultural leaders; Choctaw elders; research team; elders; hula members; teachers; community-based organizations; investigations from the University of Hawaii and Washington state; health providers; housing representatives; environmental departments; cultural leaders; knowledge keepers; youth; parents	Implementation strategies, indigenous worldviews, and protocols derived from five diverse community-based Native health intervention studies	Culturally grounded models of health promotion: original instructions; relational restoration; narrative-embodied transformation; and indigenous CBPR
21	Young (2017) [32] (Canada)	Case Study	15 Canadian Aboriginal communities	50 Canadian Aboriginal communities	Aboriginal children	Planning discussions on challenges and best practices to implement a children’s well-being assessment tool	None

*Ind, individual; Inter, interpersonal; Org, organizational; Comm, community; Soc/Pol, society/policy

**Level of SEM per Barrier Category: Social determinants of health in communities = Community/Society-Policy; Personnel Challenges & High Turnover = Organizational; Funding = Organizational; Lack of Integration with Cultural Values = Organizational/Community; Limited Retention and High Attritio*n* = Intrapersonal/Organizational; Distrust = Intrapersonal/Interpersonal/Organizational; Technology Barriers = Organizational; Insufficient Evaluation Skills = Intrapersonal/Organizational; Climate Conditions = Intrapersonal/Organizational/Community/Society-Policy

**Table 3:** Barriers classified based on the socio-ecological model (SEM) and barrier category themes

Study (year)	Barriers (*n* = 100)	Socio-ecological model (SEM) level*						Barrier category**							
		Ind	Inter	Org	Comm	Soc/Pol	Social determinants of health in communities	Personnel challenges and high turnover	Funding	Lack of integration with cultural values	Limited retention and high attrition	Technology barriers	Distrust	Insufficient evaluation skills	Climate conditions
Barlow (2018) [16]	Socioeconomic, geographic, and structural challenges	X			X	X	X		X						X
	Poverty, economic, and human resource challenges that strain home-visiting implementation	X		X	X	X	X	X	X						
	Lack of reliable vehicles to drive to homes and implement intervention			X			X								
	Complex issues of historical oppression and trauma that burden families		X		X	X	X								
	Homelessness as a serious challenge for clients and their “home visitors”			X	X		X	X							
Black (2018) [17]	Insufficient broadband			X								X			
	Poorly maintained computers			X								X			
	Financial Instability			X					X						
	Loss of interest in the program and attrition	X									X				
Jernigan (2020) [8]	None														
Jernigan (2016) [20]	Inability to compare readiness scores across different stakeholder groups			X			X			X					
	Community members identifying themselves as members of multiple stakeholder groups				X		X								
	Changes in program leadership			X				X							
	Changes in funding support			X					X						
	Limited resources influencing readiness levels			X	X			X							
Counil (2012) [13]	Isolation from food production and distribution centers	X			X		X								
	Communities isolated from each other				X		X								
	Extreme climate weather conditions					X									X
	Cost of transportation	X		X		X	X		X						
	High price of imported goods	X				X	X		X						
	High costs of healthcare professionals and health promotion campaigns			X				X	X						
	High turnover of healthcare professionals, store managers, and volunteers			X				X							
	Risk of food insecurity in community				X	X	X								
	Clash of dietary cultures					X				X					
	Lack of language-sensitive and culturally sensitive dietary recommendations					X				X					
	Sedentary settlement due to school, trading posts, and other governmental incentives	X			X	X	X								
	Structural violence					X	X								
Craig Rushing (2018) [12]	Infrastructure shortcomings (internet connection; mobile broadband use)			X		X						X			
	Low funding for the network of technical assistance			X					X						
	Lack of funding to host kick-off events to build community awareness			X	X				X						
	Lack of funding to secure approval from local tribal communities			X	X				X						
Douglas (2013) [18]	Contextual barriers to knowledge use including individual health (comorbidities)	X					X								
	Lack of proper diagnosis within the healthcare system			X			X								
	Low funding levels at the level of the health system			X	X	X	X		X						
	Competing healthcare staff demands			X				X							
	Strain of acute care on health system			X	X	X	X								
	Access to care in remote areas	X			X		X								
	Childcare when in need of healthcare services	X		X			X								
	Negative healthcare experiences	X					X	X							
	Capacity of family to respond to healthcare stressors		X				X	X					X		
	Capacity of schools to respond to stress, variety of caregivers, and socioeconomic factors			X			X	X	X				X		
	Capacity of community to respond to stress, variety of caregivers, and socioeconomic factors				X		X	X	X				X		
	Lack of asthma awareness and low reading levels	X			X		X								
Gates (2013) [19]	Challenges to improved dietary intakes and sustainability in the first year			X	X		X								
Jiang (2013) [21]	Skepticism of grantee staff about the importance and success of evaluation			X				X						X	
	Staff had no experience in evaluating other rigorous programs			X				X						X	
	Challenge of participant retention	X									X				
	Scheduling difficulties	X						X			X				
	Participants moving away	X									X				
	Compromised attendance of participants due to stressful lifestyles	X			X						X				
	Challenge to sustain intervention effects for long periods of time	X			X	X		X		X	X				
Kaufman (2018) [22]	Integration of new routines into settings often imbued with particular cultural expectations of care and service			X	X		X			X					
	Limited financial and material resources			X	X			X	X						
Markham (2016) [10]	Frozen screens (4/6 programs)	X		X								X			
	Long loading time of activities			X								X			
	Trouble navigating programs	X										X			
	Technical and connectivity issues at sites			X								X			
Martindale-Adams (2017) [23]	Staff concern about identification of caregivers in cases of loss of memory		X	X			X	X							
	Lack of awareness of public health nurses about patient memory concerns			X				X							
	Family members not identifying themselves as caregivers		X				X								
Mokuau (2008) [24]	None														
Moleta (2017) [25]	Short duration of staff training for the amount of material covered			X				X							
	Limited information on alternative and traditional medicine practices			X				X							
	Limited strategies to help uninsured clients			X		X		X							
Nadin (2018) [26]	Limited funding for palliative care and community care services			X		X			X						
	Lack of service delivery funds			X					X						
	Lack of housing infrastructure and overcrowding				X		X								
	Difficulty in assessing system-level outcomes					X	X								
Orians (2004) [15]	Limited experiences of tribes in providing and participating in federally funded health promotion and disease prevention programs			X	X			X	X	X					
	Limited resources for chronic disease care			X	X	X	X	X	X						
	Inadequate mammography services			X	X	X	X								
Pei (2019) [28]	Lack of community awareness about fetal alcohol spectrum disorder				X		X								
	Stigma around the disease				X	X				X					
	Reluctance of women to admit using substances	X											X		
	Complex needs of clients served by Parent-Child Assistance Program		X	X			X								
Rasmus (2019) [29]	None														
Short (2014) [30]	Lack of integration of specific cultural and contextual variables of a given community				X					X					
	Timing of the intervention	X		X	X						X				
	Lack of integration of local customs and cultural values into program activities			X	X					X					
	Having no tribal police department and a secondary enforcement law				X	X	X	X							
	Shortage of police officers				X	X		X							
	High turnover in police chief positions			X	X	X		X							
	Large geographic distance between the community and the evaluation team				X		X	X							
	Limitations in evaluating community outcomes				X									X	
	Conflicts in scheduling community meetings				X						X				
Walters (2020) [31]	None														
Young (2017) [32]	Communication differences	X		X	X					X					
	Capacity/turn-over			X				X							
	Building trust over distance				X								X		
	Negative historical experiences with research				X	X				X			X		
	Local complexities				X	X	X								
	Multiple service providers		X	X				X							
	Timeline uncertainties			X							X				
	Total	22	6	49	41	26	38	29	18	11	9	7	6	3	2

*Ind, individual; *Inter*, interpersonal; *Org*, organizational; *Comm*, community; *Soc/Pol*, society/policy

**Level of SEM per Barrier Category: Social determinants of health in communities = Community/Society-Policy; Personnel Challenges & High Turnover = Organizational; Funding = Organizational; Lack of Integration with Cultural Values = Organizational/Community; Limited Retention and High Attrition = Intrapersonal/Organizational; Distrust = Intrapersonal/Interpersonal/Organizational; Technology Barriers = Organizational; Insufficient Evaluation Skills = Intrapersonal/Organizational; Climate Conditions = Intrapersonal/Organizational/Community/Society-Policy

**Table 4:** SISTER-Strategies by domain, rank, and percentage of citation

#^a^	Strategy	Domain^d^	Rank	Strat. (%)	Imp.^b^	Feas.^c^
21	Build partnerships (i.e., coalitions) to support implementation	Develop stakeholder interrelationships	1	86		
22	Capture and share local knowledge	Develop stakeholder interrelationships	2	81		x
17	Tailor strategies	Adapt and tailor to context	3	71		
23	Conduct local consensus discussions	Develop stakeholder interrelationships	4	52		
37	Conduct educational meetings	Train and educate stakeholders	5	38		
9	Monitor the progress of the implementation effort	Use evaluative and iterative strategies	5	38	x	
57	Involve students, family members, and other staff	Engage consumers	5	38		
39	Conduct ongoing training	Train and educate stakeholders	5	38	x	
35	Use advisory boards and workgroups	Develop stakeholder interrelationships	6	33		
43	Make training dynamic	Train and educate stakeholders	6	33	x	x
28	Inform local opinion leaders	Develop stakeholder interrelationships	7	29		
24	Develop academic partnerships	Develop stakeholder interrelationships	7	29		
42	Distribute educational materials	Train and educate stakeholders	7	29		x
40	Create a professional learning collaborative	Train and educate stakeholders	8	24		
58	Prepare families and students to be active participants	Engage consumers	8	24		
13	Peer-assisted learning	Provide interactive assistance	8	24		
14	Provide practice-specific supervision	Provide interactive assistance	8	24		
12	Facilitation/problem-solving	Provide interactive assistance	9	19		x
15	Provide local technical assistance	Provide interactive assistance	9	19		
16	Promote adaptability	Adapt and tailor to context	9	19		
29	Involve governing organizations	Develop stakeholder interrelationships	9	19		
44	Provide ongoing consultation/coaching	Train and educate stakeholders	9	19	x	
1	Assess for readiness and identify barriers and facilitators	Use evaluative and iterative strategies	9	19		
7	Develop instruments to monitor and evaluate core components of the innovation/new practice	Use evaluative and iterative strategies	9	19		
34	Recruit, designate, train for leadership	Develop stakeholder interrelationships	9	19		
68	Change/alter environment	Change infrastructure	9	19		

^a^SISTER category number based on Cook et al., 2019 [38]. A total of 26 strategies are documented in the table. The rationale behind the cut-off is that the strategy has been included in at least four out of the twenty-three studies

^b^Ranked as highly important by Lyon et al., 2019 [33]

^c^Ranked as highly feasible by Lyon et al., 2019 [33]

^d^All 9 SISTER domains were cited (Cook et al, 2019 [38]). They numbered (from highest to lowest) based on the 26 (out of 60) highly ranked SISTER strategies (≥ 4 studies) cited within seven of these domains: Develop stakeholder interrelationships (31%); Train and educate stakeholders (23%); Provide interactive assistance (15%); Use evaluative and iterative strategies (12%); Adapt and tailor to context (8%); Engage consumers (8%); and Change infrastructure (4%). The remaining two domains (“Support educators” and “Use financial strategies”) included strategies cited in less than four studies and were thus not included in the table

## Results

The initial study extraction resulted in 79,585 studies from PubMed (*n* = 87), EMBASE (*n* = 79,485), and Medline Ovid (*n* = 13) (Fig. 1). Studies were excluded due to targeting non-Native communities (*n* = 89), implementing medical protocols and treatments (*n* = 79,398), taking place outside the USA or Canada (*n* = 17), or failing to address dissemination or implementation processes (strategies, theories, or frameworks) related to evidence-based or evidence-informed programs among Indigenous communities (*n* = 21). Duplicate studies were deleted (*n* = 16). Thirty-eight studies met inclusion criteria from PubMed (*n* = 19), EMBASE (*n* = 18), and Medline (*n* = 1). An additional 17 studies were excluded following a full study review due to failure to 1) report D&I strategies (*n* = 2), 2) correspond to definitions of D&I (*n* = 8), or 3) focus on D&I (*n* = 7). A total of 21 eligible studies were retained for analysis.

The 21 retained studies were published between 2004 and 2020 (Table 2). Most studies (14/21, 66%) were published in 2015 or later (*n* = 14), and most were conducted in the USA (14/21, 66%). Study designs included qualitative studies (*n* = 3); case studies (*n* = 7); randomized controlled trials (*n* = 3); pilot studies (*n* = 2); cross-sectional studies (*n* = 2); quasi-experimental studies (*n* = 3); and systematic review (*n* = 1) Study implementation duration varied from 5-hour trainings to projects of 13 months duration. For quasi-experimental studies and randomized controlled trials, study follow-up periods ranged from 0 months (assessment directly after program completion) to 3 years. The evidence-based programs described in the studies were community-based programs carried out in diverse tribal settings.

### Priority populations and key stakeholders

Priority populations who were actively involved (or targeted) in implementation activities were adults (81%, *n* = 17) and/or children/youth (43%, *n* = 9) (Table 2). Adult participants included tribal members and elders (AI/AN, *n* = 4; NH, *n* = 1; First Nation, *n* = 1), community health workers (*n* = 1), women (AI/AN, *n* = 1; Choctaw, *n* = 1), mothers and caregivers (AI/AN, *n* = 1; First Nation, *n* = 1, Choctaw, *n* = 1); and those with chronic disease and health challenges (AI/AN with Alzheimer’s, *n* = 1; adults enrolled in fetal alcohol spectrum disorder services, *n* = 1; Indigenous victims of car accidents, *n* = 1; NH with cardiovascular disease and hypertension, *n* = 2). Key stakeholders who were crucial to planning program implementation included decision makers in healthcare, school, community, organizations, academics, and government (Table 2).

### Content domains

The evidence-based programs targeted a variety of health domains, including chronic disease and injury, substance misuse, wellness and illness prevention, and historical trauma (Table 2). Chronic disease and injury topics included hypertension and cardiovascular disease (*n* = 3), obesity (*n* = 1), asthma (*n* = 1), diabetes (*n* = 1), hearing loss (*n* = 1), Alzheimer’s (*n* = 1), palliative care (*n* = 1), and motor vehicle crashes (*n* = 1). Substance misuse included misuse of alcohol and other drugs (*n* = 5) and tobacco use (*n* = 1). Wellness and illness prevention topics included maternal and child health (*n* = 1), sexual health (*n* = 4), nutrition (*n* = 4), physical activity (*n* = 1), improved access to healthcare services (*n* = 2), breast and cervical cancer screening (*n* = 1), overall children’s well-being (*n* = 1), and reduction of environmental contaminants exposures (*n* = 1). One study focused on a historical approach to health through walking the Trail of Tears and 2 studies reported programs addressing multiple health topics [8, 10, 31].

### Tribal communities and settings

Diverse tribal communities were represented in this review, including AI/AN (*n* = 13), Inuit (*n* = 2), and First Nation/Indigenous (*n* = 7), and Native Hawaiian (*n* = 2) communities (Table 2). AI/AN communities included tribes in Oklahoma, California, Alaska, Arizona, and the Pacific Northwest (Oregon, Idaho, and Washington). Inuit communities included tribes in Greenland and Northern Canada. First Nation/Indigenous and Native Hawaiian communities had representation from multiple regions in Canada and Hawaii respectively. Settings comprised Native nations, reservations and reserves, tribal agencies and associations, health agencies, academic affiliates, and schools (Table 2).

### D&I barriers

Eighty-nine barriers to implementation were reported in 17 studies (81%), representing the five levels of the socio-ecological model (SEM): Individual (*n* = 22), interpersonal (*n* = 6), organizational (*n* = 49), community (*n* = 41), and society/policy (*n* = 26) (Table 3). Barriers were also sorted into nine categories (Table 3) based on major themes that were established through similarity of barriers highlighted across studies at the different levels of SEM. Some barriers fit into the SEM levels, and thus generated more than one theme. For instance, Barlow et al. (2018) highlighted “socioeconomic, geographic, and structural challenges” as a barrier, affecting the individual, community, and society/policy levels of the SEM. The barrier category themes emerging from this barrier and its subsequent SEM classification included “funding,” “social determinants of health in communities,” and “climate conditions.” Most cited barriers (*n* = 38) sorted into the Community/Society-Policy category of “Social determinants of health in communities.” A majority of studies also cited “Personnel challenges and high turnover” (*n* = 29), “Funding” (*n* = 18); “Lack of integration with cultural values (*n* = 11), and “Limited retention and high attrition” (*n* = 9) Other barrier categories included Technology barriers (*n* = 7); Distrust (*n* = 6); Insufficient evaluation skills (*n* = 3); and Climate conditions (*n* = 2).

### D&I models

Sixteen studies (76%) used a specific D&I model to promote the adoption and implementation of health promotion EBIs in Indigenous communities (Table 2). Eight different unique models were cited. Community-Based Participatory Research (CBPR) was most commonly reported (*n* = 11). Four studies used models that focused on dissemination and/or implementation (Knowledge-to-Action Framework, Diffusion of Innovation Theory, and RE-AIM), andragogy (Adult Learning Theory), or inductive and culturally responsive processes (Culturally Grounded Models of Health Promotion). Remaining models focused on the broader implementation process inclusive of dissemination. Ten studies used a D&I model for the purpose of identifying barriers and/or facilitators to the dissemination process; seven studies highlighted the main barriers and/or facilitators that were encountered during the implementation process.

### Implementation strategies

All SISTER domains were represented, and all extracted D&I strategies were matched to relevant SISTER strategies However, not all SISTER strategies were represented in the included studies. One hundred and eighty-four D&I strategies (*n* = 184) were identified, corresponding to 60 (80%) of the SISTER strategies. A range of three through nineteen strategies were reported in any one study. The most commonly reported SISTER strategy (identified in 86% of studies) was: “Build partnerships (i.e., coalitions) to support implementation” (#21) (Table 4). Four SISTER strategies, previously recognized as being *highly important* for D&I success were represented in the top 10 strategies [33]. These were “Conduct ongoing training” (#39), “Monitor the progress of the implementation effort” (#9), “Provide ongoing consultation/coaching” (#44), and “Make training dynamic” (#43). These strategies occur in the domains of “Train and educate stakeholders” and “Use evaluative and iterative strategies.” Four SISTER strategies previously described as *most feasible* for successful D&I were also represented in the top 10. These were: “Make training dynamic” (#43), “Distribute educational materials” (#42), “Facilitation/Problem solving” (#12), and “Capture and share local knowledge” (#22) (Table 4).

## Discussion

The purpose of this scoping review was to identify barriers and mitigating D&I processes related to the adoption and implementation of EBIs in Indigenous communities. Analysis of the 23 included studies (conducted between 2004 and 2020) may contribute to our understanding of common barriers and mitigating D&I models and strategies used to successfully disseminate and implement EBIs in Indigenous communities in the United States, Hawaii, Pacific Islands, and Canada [8, 10, 12–32].

### D&I models

The majority of the studies (76%) used a D&I model to guide the dissemination and/or implementation of an EBI. Such studies have increased in recent years with 66% of the included studies published since 2015. This reflects the recognition of D&I to address existing and emerging health disparities and is consistent with a broader increase in D&I research. The most frequently reported model was Community-Based Participatory Research (CBPR) (*n* = 11), which encompasses an array of principles consistent with partnering with Indigenous minorities [70]. A recent systematic review by Julian McFarlane et al. (2021) [71] highlighted the large increase in the number of CBPR-related studies targeting a broad racial and ethnic representation in research. More than 85% of these studies saw statistically positive outcomes when applying CBPR methods, particularly community partner participation in study advisory committees, data collection, the development of interventions, and participant recruitment [65].

CBPR aims to (1) recognize the Indigenous community as a unit of identity, (2) build on the community’s strengths and resources, (3) facilitate collaborative partnerships in all phases of the research, (4) integrate local knowledge and actions that benefit all partners, (5) empower community members to address social inequalities, (6) involve a cyclical and iterative process, (7) address health from both positive and ecological perspectives, and (8) disseminate findings and knowledge gained to all partners [72]. These principles represent an important foundation to guide ethical D&I studies and are complementary with common reported strategies (described below). Yet CBPR is not without limitations and may not account for the specific array of facilitation strategies and prescriptive steps associated with many D&I models [70]. The frequency of application of D&I models other than CBPR was relatively low (n = 5). Greater research on D&I models in Indigenous communities may enhance the quality of implementation planning and evaluation in those settings, building empirical evidence for the utility of such models using traditional CBPR approaches [73, 74]. Encouraging these systematic approaches can also expand our knowledge-base on the most salient D&I models and strategies for Indigenous communities [73, 74].

### Barriers and mitigating D&I strategies

This study reinforced the critical need to identify and implement D&I strategies at all levels of the socio-ecological model to address common barriers that impede implementation efforts. The social milieu in which programs are deployed in Indigenous communities can be complex and challenging. Principal among these challenges are consideration of social determinants of health, perceptions of community trust, community skill sets, and financial challenges. Social determinants of health are important considerations when attempting to reach underserved populations as they address issues related to the complex mental, health, social, physical, and socioeconomic issues of communities. They can represent major barriers to program implementation. Cited factors that can compromise program implementation in Indigenous communities include poverty, homelessness or residential instability, geographic remoteness with accompanying challenges of access to healthcare service, and greater transportation expenses. Across the literature, intentional information gathering and community involvement were critical to program success. These included “assessing for readiness and identifying barriers and facilitators,” “involving governing organizations,” “informing local opinion leaders,” and “involving students, family members, and other staff” [13–18, 23, 24, 26–31]. More broadly, the strategy of “changing or altering the environment” was employed where feasible, again in consort with community stakeholders.

Complicating the challenge of social determinants is the perception of trust between community members and healthcare providers, or between program participants and the entity delivering the program (i.e. organization, academic institution, governmental agency). These relate to the barrier categories of “distrust” and “lack of integration with cultural values.” Building partnerships to support implementation was the most commonly cited SISTER strategy across the included studies (86%). However, despite the importance of building partnerships in the community and sharing its local knowledge, additional strategies are indicated. Most studies (55%) reported organizational barriers related to involving the views and experiences of elders, community health workers, families, and youth as part of the implementation process [13, 15, 20–22, 28, 30, 32]. Hearing the community voice and attending to community needs can further engender trust. The expertise of Indigenous community members, elders, and health planners, many of whom have unique skills, particularly in the fields of cultural adaptation, tailoring interventions, and appropriate implementation is highly valued and can help to alleviate community concerns [75] as well as smooth logistics involved with navigating the complex tribal internal review and research review boards necessary for collaboration with external academic and research partners [8].

The studies mentioned other D&I strategies that can promote cohesion around program implementation at the organizational level. These included recruiting and retaining families through trust-building; ensuring convenience of program offerings, forming local advisory boards and task forces, creating cultural activities, and using mass media tools (newspaper, written materials, and radio programs) to promote programs. Organizational administration included attention to data management; capacity-building efforts, prioritization of strategies, and collaboration with academic researchers and regional stakeholders [8, 10, 12–32]. Frequently cited was the need to elicit community support through engagement of the community and Native stakeholders in the planning and implementation process [13, 15, 20–22, 27, 28, 30, 32]. This is vital to aid in cultural learning, integration of cultural values, and inclusion of indigenous role models to optimize cultural compatibility and the potential for sustained implementation. Native stakeholders should be engaged in the planning phase to ensure that their needs and desires are fulfilled [13, 15, 20–22, 28, 30, 32].

Staff training, personalized technical assistance, staff commitment to engage youth, and continuous evaluation of staff performance [8, 10, 12, 17, 23–25, 29, 30] are necessary for sustained implementation of programs within Indigenous communities. These strategies can mitigate the “Personnel Challenges and High Turnover” that was cited in 65% of the studies [13–16, 18, 20–23, 25, 32]. High turnover rates can undermine personnel skills training due to the continuous loss of acquired talent and the need to accustom new personnel to the community and program material [13–16, 18, 20–23, 25, 32]. Insufficient skills needed to deliver the program material were cited as a common barrier. SISTER strategies included under the two domains—“training and educating stakeholders” and “developing stakeholder interrelationships”—could help address those common barriers.

Funding is a continuous challenge affecting sustained implementation. Funding issues were frequently reported by Native stakeholders during interviews and focus group sessions and emerged as a main theme in qualitative studies [13–15]. This included a lack of sustained funding at the organizational level to increase research outputs [12–18, 20, 22, 26]. This in turn led to a limited availability of resources and thus the inability to maintain programs outcomes for longer periods of time. Specific financial barriers included high cost of salaries, housing, transportation, and other mission fees needed to hire social workers, program adopters and implementers, and healthcare workers [13, 14, 20–23, 25, 32]. Accessing new funding sources was a leading D&I strategy employed in Native communities. Continuous delivery of program resources and material is predicated on sustained financial support without which D&I efforts are hobbled [55].

Studies describing intervention implementation at the policy level cited the importance of creating and implementing new public health policies to overcome societal and economic barriers. These crosscut other socioecological levels and included the high costs of imported goods and healthy foods, inadequate funding allocations to healthcare systems, limited assistance for uninsured clients, limited resources for chronic diseases, improper management of historical oppression and trauma, infrastructure shortcomings, and high levels of poverty [13, 15, 16, 18, 25]. All nine domains encompassing multiple SISTER strategies were mentioned in the studies. Studies on the effectiveness of D&I strategies in this domain are limited [54, 61]. Future work could focus on the multilevel policies that shape social determinants of health and their impact on D&I outcomes in Indigenous settings. Holistic approaches with culturally tailored strategies are essential to overcome potential barriers.

### Strengths & limitations

These studies correspond highly to reported SISTER strategies previously categorized as important and feasible in non-indigenous contexts [61]. Four of five strategies rated as *most important* were among the top ten reported in this review. These strategies included (1) “Monitor the progress of the implementation effort” (#9); (2) “Conduct ongoing training” (#39); (3) “Make training dynamic” (#43); and (4) “Provide ongoing consultation/coaching” (#44). The 5^th^ strategy, “Improve implementers’ buy-in” (#51), was not represented. Four of five strategies rated as *most feasible* were among the top ten reported in this review. These included (1) “Capture and share local knowledge” (#22), (2) Distribute educational materials” (#42); (3) “Make training dynamic” (#43); and (4) “Facilitation/Problem solving” (#12). The 5^th^ strategy, “Remind school personnel” (#53), was not represented in any of the studies. Financial strategies categorized under the domain “Use financial strategies” received a low feasibility rating in Lyon et al. (2019) and were only reported in a few of our studies [61]. This may reflect the lack of funding that was identified as a barrier in 50% of the studies [61].

Findings need to be interpreted in the context of study limitations. First, despite a comprehensive search of the most relevant psychosocial databases, this review did not include tracing of reference lists in included studies, hand-searches of journals, or grey literature. Broader reviews are recommended that account for these sources. Second, the D&I field is growing rapidly, so it is possible that some relevant studies were not found due to inadvertent omission of search terms. The mesh terms included as many technical D&I keywords as possible and the collaboration of a research librarian who imposed rigor in the protocols likely mitigated this concern. Future reviews are recommended to include emerging terms from this rapidly evolving field. Third, the scope of the current review was limited. Formal assessment of the quality of the included studies was beyond scope and the inter-rater reliability, though acceptable with domain and strategy correspondence of 70% and 90% respectively, was based on assessment of only eight (38%) of the included studies. Fourth, matching the identified D&I strategies to the SISTER strategies was challenging due to the diversity of terms used to describe any given strategy. Consistency of terminology represents a challenge for any emerging field. Standardizing the nomenclature will be important to enable clear research and practice guidelines for EBI implementation. Fifth, the use of SEM to categorize barriers and contextual factors limits comparison to other D&I frameworks such as CFIR (Consolidated Framework for Implementation Research) or EPIS (Exploration, Preparation, Implementation, Sustainment). However, SEM categorization will inform the selection of multilevel implementation strategies to facilitate EBI uptake in Indigenous communities [52]. It also provides an objective assessment agnostic of any particular D&I framework [52]. Finally, the SISTER strategies were originally developed based on studies in non-Indigenous settings. Although the taxonomy is comprehensive and provides a useful comparison for non-indigenous settings, it may also miss cultural influences or D&I processes that are unique to Indigenous communities. The similarity with findings from Lyon et al. (2019) indicates some validity across cultural settings [61]. Future studies are recommended to provide guidance on which strategies to use to promote behavior and health changes in Indigenous settings. The use of existing accepted taxonomies in this study may provide guidance for future work.

## Conclusion

This scoping review describes D&I efforts to translate research and change practice in Indigenous communities across the USA and Canada. Results may contribute to a broader perspective of barriers and mitigating strategies to inform and guide future D&I initiatives in Indigenous communities, with a goal to reduce health disparities in these populations. This study emphasized ranks of barriers and related D&I strategies (matched to the adapted SISTER strategies) that appear salient for Indigenous communities including focusing on culturally relevant partnerships, trainings, evaluations, and adaptation. The existing diversity in culture, beliefs, values, and resources across tribes and borders is a major consideration for future D&I initiatives. Efforts to apply D&I models and strategies are increasing within Native communities as they are in non-indigenous communities. This study can guide researchers and community partners using D&I models and strategies to improve the reach and sustainability of evidence-based programs in Indigenous communities.

## Supplementary Information

**Additional file 1.**

## Data Availability

All data generated or analyzed during this study are included in this published article [and its supplementary information files].
